# The Combination of Mitragynine and Morphine Prevents the Development of Morphine Tolerance in Mice

**DOI:** 10.3390/molecules18010666

**Published:** 2013-01-04

**Authors:** Sharida Fakurazi, Shamima Abdul Rahman, Mohamad Taufik Hidayat, Hairuszah Ithnin, Mohamad Aris Mohd Moklas, Palanisamy Arulselvan

**Affiliations:** 1 Department of Human Anatomy, Faculty of Medicine and Health Sciences, Universiti Putra Malaysia, 43400 UPM Serdang, Selangor, Malaysia; E-Mails: taufik@medic.upm.edu.my (M.T.H.); aris@medic.upm.edu.my (M.A.M.M.); 2 Laboratory of Vaccines and Immunotherapeutics, Institute of Bioscience, Universiti Putra Malaysia, 43400 UPM Serdang, Selangor, Malaysia; E-Mail: arulbio@gmail.com; 3 Faculty of Pharmacy, Cyberjaya University College of Medical Sciences, 63000 Cyberjaya, Selangor, Malaysia; E-Mail: alfaqirah_msc@yahoo.com; 4 Department of Pathology, Faculty of Medicine and Health Sciences, Universiti Putra Malaysia, 43400 UPM Serdang, Selangor, Malaysia; E-Mail: hairusza@medic.upm.edu.my

**Keywords:** antinociceptive, cAMP, *Mitragyna speciosa*, morphine, tolerance

## Abstract

Mitragynine (MG) is the major active alkaloid found in *Mitragyna speciosa* Korth. In the present study, we investigated the enhancement of analgesic action of MG when combined with morphine and the effect of the combination on the development of tolerance towards morphine. Mice were administered intraperitoneally with a dose of MG (15 and 25 mg/kg b.wt) combined with morphine (5 mg/kg b.wt) respectively for 9 days. The antinociceptive effect was evaluated by a hot plate test. The protein expression of cyclic adenosine monophosphate (cAMP) and cAMP response element binding (CREB) was analyzed by immunoblot. Toxicological parameters especially liver and kidney function tests were assessed after the combination treatment with MG and morphine. The concurrent administration of MG and morphine showed significant (*p* < 0.05) increase in latency time when compared to morphine alone group and the outstanding analgesic effects in the combination regimens were maintained until day 9. For the protein expression, there was a significant increment of cAMP and CREB levels (*p* < 0.05) in group treated with 5 mg/kg morphine but there was no significant change of these protein expressions when MG was combined with morphine. There was a significant changes in toxicological parameters of various treated groups. The combination treatment of MG and morphine effectively reduce the tolerance due to the chronic administration of morphine.

## 1. Introduction

The management of chronic pain is one of the greatest challenges in modern medicine. Millions of people around the World suffer from a variety of causes leading to chronic pain such as arthritis, cancer and nerve injury, *etc*. Opiates such as morphine have been used to treat various kinds of pain for centuries [1]. Its long term use of morphine is very limited due to unwanted side-effects and among the side-effects of morphine, tolerance and dependence are the hardest to counteract.

Currently, a number of natural active compounds have been detected to have potential analgesic effects [2,3]. One of these natural compounds is mitragynine which has been isolated from *Mitragyna speciosa* Korth. *Mitragyna speciosa* is a plant that is most abundantly found in Malaysia and Thailand which popularly known as ‘ketum’ in Malaysia and ‘kratom’ in Thailand and it has been used as a herbal drug for decades. Traditionally, the *Mitragyna speciosa* was used to alleviate pain, diarrhea, hypertension, cough, and as a substitute for morphine in treating addicts [4,5].

**Figure 1:**
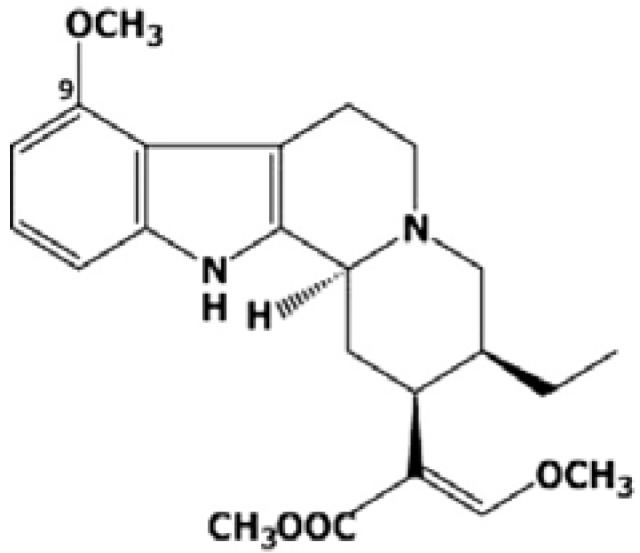
Structure of mitragynine.

Over 25 alkaloids have been isolated from this traditional plant [6] and an alkaloids, mitragynine was obtained as the major phyto-constituent (66.2%) together with its other analogues. Mitragynine (MG, Figure 1) has an indole structure, with its 4th position substituted by a methoxy group. It has a 9-methoxy-corynantheidine molecular structure with the molecular weight of 398.50 (C_23_H_30_N_2_O_4_) [4]. Numerous studies have been made either on the major constituent MG [7] as well as other related alkaloids [8,9] and it was found that these compounds have opioid-like activities. 

Mitragynine was also reported to be comparable to codeine as an analgesic [10,11] and researchers found that MG is an opioid receptor agonist natural compound [12]. Intracerebroventricular (i.c.v) and intraperitoneal (i.p) administration of MG exerted a potential antinociceptive effect on mechanical and thermal noxious stimuli [13]. Various pharmacological investigations of MG have also revealed that it has an antinociceptive action through the supraspinal opioid receptors when the effect of MG was antagonized by i.c.v naloxone [13]. Its action is dominantly mediated by μ- and δ-receptor subtypes. MG only blocked the effect of Nor-binaltorphimine, a selective κ-opioid receptor antagonist in the tail-pinch test but not in the hot-plate test [14]. Recently, we reported that MG has strong antinociceptive properties through supraspinal opioid receptor systems and these studies were confirmed by various specific antagonists [15].

Previous studies have suggested that the use of opioid combinations may improve analgesia, reducing the toxicity and delaying the development of tolerance [16,17]. Tolerance to analgesics means the diminishing effect of analgesics due to various physiological adaptations following repeated administration of the medications. In order to obtain similar analgesic effect, the dose of the opioid is increased to overcome any tolerance that may develop. Besides, combination of opioids with other classes of analgesics, especially from natural sources, may reduce the sensitization processes and optimize pain therapy [18]. Thus, the combinations of medications that offer analgesic synergism would allow the reduction of required dosage which gives optimum analgesic effects with low incidence of tolerance is needed. 

Opiates, acting on opioids receptors via G proteins, inhibit cyclic AMP (cAMP) formation [19,20] and this result in inhibition of neuronal excitability and synaptic transmission mediated via cAMP and it represents a cellular mechanism of opioid action. Besides, cAMP also regulates the expression of specific genes via a conserved gene promoter element CRE (cAMP response element) [21]. The cAMP response element binding (CREB) is a transcription factor that binds to CRE in the promoter region of target genes and modulates their expression [22,23]. In continuation of opioid exposure, activity of cAMP gradually increases which leads to tolerance of cAMP transmission at the single cell level. It was also related that the alterations in CREB expression following morphine treatment were caused by the cAMP pathway activation [24,25]. Furthermore, the activation and increased expressions of CREB levels will also relates to the development of tolerance [26,27].

Hence, the present study was designed to investigate the analgesic action of combinational treatments of mitragynine and morphine. In this regard we evaluated the tolerance development in morphine treated animals in the presence of natural compound, mitragynine. The toxicological effects of the combinational treatments were also evaluated to confirm the non-toxic nature combinational approach.

## 2. Results and Discussion

### 2.1. Results

#### 2.1.1. Antinociceptive Effect of Combinational Treatment of MG and Morphine

**Figure 2:**
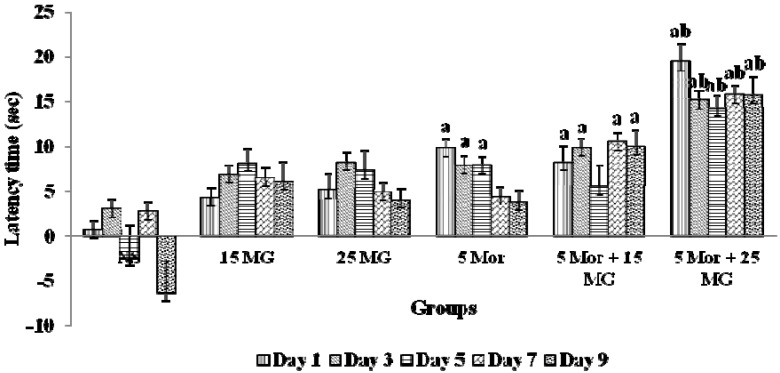
The effects of control group [Normal saline (NS), mitragynine (MG) (15 mg/kg, 25 mg/kg], morphine (5 mg/kg) and combination groups (5 mg/kg Mor + 15 mg/kg MG; 5 mg/kg Mor + 25 mg/kg MG) on latency time using hot-plate test for 9 days treatments. Each column represents the mean ± SEM of latency time (s) of seven animals in each group. Values are statistically siginficant at *p* < 0.05. a: Morphine and combination treated groups when compared with control group and b: Combination treated group when compared with morphine alone (*p* < 0.05).

Figure 2 summarizes the results of latency time showed by the test animals when treatment of mitragynine was combined with morphine. The test mice treated with morphine showed a high antinociceptive effect on day 1 when compared to the control and mitragynine treated groups. The latency time was significantly reduced on day 5 and the same effect was seen subsequently until the end of the experiemnt on day 9. Meanwhile, when the animals were treated with mitragynine alone, the latency time was also reduced. Intriguingly, when mitragynine and morphine were administered concomitantly, the latency time was improved. These findings suggest that the mice experiencing analgesic effect for a longer duration compared to the treatment of either morphine or mitragynine alone. 

#### 2.1.2. The Effects of MG and Morphine on the Expression of cAMP

**Figure 3:**
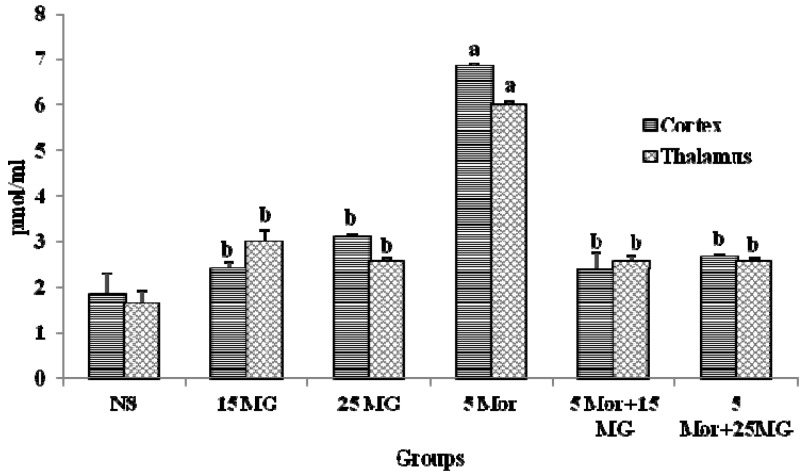
The expression of cAMP protein in thalamus and cortex of control group (normal saline), mitragynine (15 mg/kg, 25 mg/kg), morphine (5 mg/kg) and combination groups (5 mg/kg Mor + 15 mg/kg; 5 mg/kg Mor + 25 mg/kg) for 9 days treatment. Each column represents expression of cAMP in pmol/ml. Values are statistically siginficant at *p* < 0.05. a: Morphine alone treated group when compared with control group and b: mitragynine and combination treated group compared with morphine alone (*p* < 0.05).

Figure 3 show the expression pattern of cAMP in all tested groups upon treatment in two parts of the brain, the thalamus and cortex. The present results demonstrated that group treated with morphine showed a significant increase in the expression of cAMP level in comparison with the control group (*p* < 0.05). Meanwhile, the level of expression of cAMP in the group treated with mitragynine and in combination with morphine was low and showed slight differences when compared to the control groups. The results shows a same pattern of cAMP expression for both the thalamus and cortex parts. These findings suggest that concomitant administration of mitragynine and morphine effectively prevents the development of tolerance following the down regulation of cAMP levels.

#### 2.1.3. The Effects of MG and Morphine on the Expression of CREB Protein

**Figure 4:**
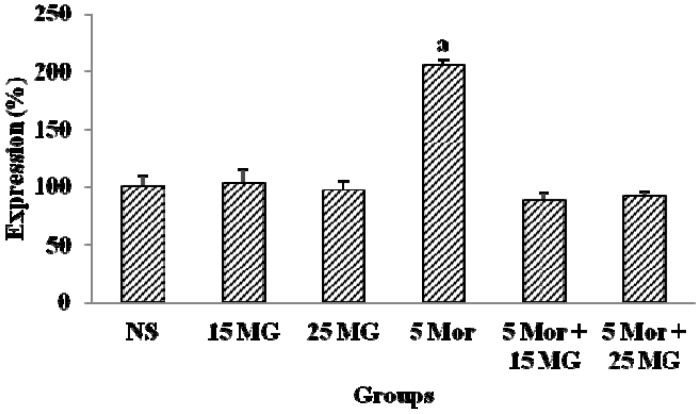
The expression of CREB protein of control group (normal saline), mitragynine (15 mg/kg, 25 mg/kg), morphine (5 mg/kg) and combination groups (5 mg/kg Mor + 15 mg/kg; 5 mg/kg Mor + 25 mg/kg) for 9 days treatment. Each column represents the percentage expression of CREB protein (n = 7). Values are statistically siginficant at *p* < 0.05. a: Morphine treated group compared with control group. Bands were detected via chemiluminescence and Image J analysis and results were expressed in percentage (%).

Figure 4 shows the expression patterns of cAMP response element binding (CREB) protein. The results demonstrated that the group treated with morphine showed a significant increase in the level of expression of CREB protein in comparison with the control group (*p* < 0.05). In the meantime, the expression of CREB protein in the group treated with mitragynine was comparatively low and it was similar to the control group. When the animals were administered with both morphine and mitragynine, the level of expression of CREB was reduced when compared to the morphine alone treated group. These findings also clearly demonstrated that combinational approach of mitragynine and morphine effectively prevents the development of tolerance. 

#### 2.1.4. The Effects of MG and Morphine on liver Enzymes

**Figure 5:**
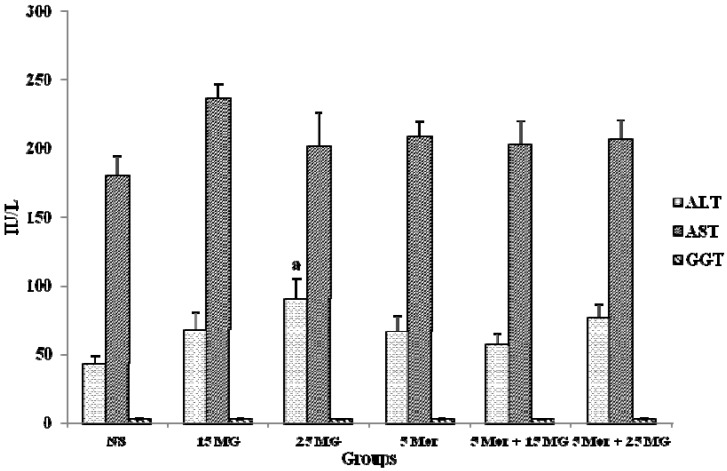
The activities of aspartate aminotransferase (AST), alanine aminotransferase (ALT) and gamma-glutamyltransferase (GGT) in control group (Normal saline), mitragynine (15 mg/kg, 25 mg/kg), morphine (5 mg/kg) and combination groups (5 mg/kg Mor + 15 mg/kg; 5 mg/kg Mor + 25 mg/kg). Each column represents the mean ± SEM of IU/L of seven animals in each group (n = 7).

Measurement of the activities of specific marker enzymes in tissues and/or fluids can be used in assessing the degree of assault and the side effects of natural/chemical compounds on organs and other vital tissues. Increased activities of liver function enzymes such as AST, ALT and GGT in the blood reflect the damage of hepatocytes which leading to hepatotoxicity. In Figure 5, there was no significant changes in liver enzymes of the treated experimental groups when compared to the control group, except for ALT in 25 mg/kg MG (*p* < 0.05). Even though there was a slight increase in the ALT level of the 5 Mor + 25 MG group, the result was not statistically significant. These results showed that combination of MG and morphine has no toxic effects on liver. 

#### 2.1.5. The Effects of MG and Morphine on Kidney Function Test

**Figure 6:**
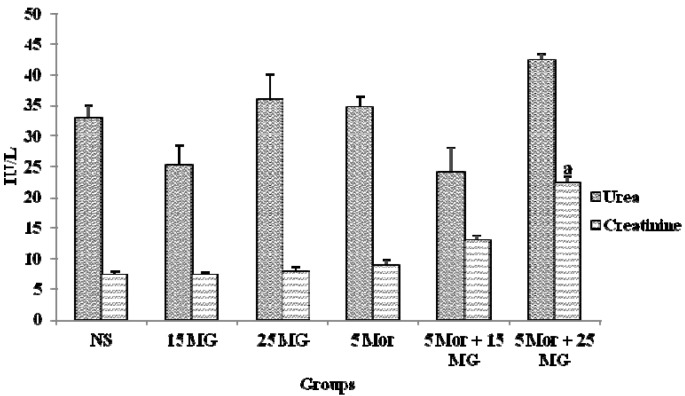
The levels of urea and creatinine in control group (normal saline), mitragynine (15 mg/kg, 25 mg/kg), morphine (5 mg/kg) and combination groups (5 mg/kg Mor + 15 mg/kg; 5 mg/kg Mor + 25 mg/kg). Each column represents the mean ± SEM of IU/L of seven animals in each group (n = 7). Values are statistically siginficant at *p* < 0.05. a: Combination treatment group compared with control group.

The levels of urea and creatinine in mice from all experimental groups are shown in Figure 6. From the results, there was no significant change in the excretion level of urea and creatinine in all treated groups when compared to the control. However, when the treatment was combined (5 Mor + 25 MG), the creatinine excretion level was significantly increased (*p* < 0.05). These results showed that higher concentration MG and morphine have minimal toxic effects on kidney. 

### 2.2. Discussion

Combination regimen is one of the alternative treatments to overcome drug induced adverse effects. The combination of two analgesic drugs has the potential to overcome tolerability, efficacy and time-to-onset limitations of the component drugs, which in certain cases, synergistically increases their analgesic properties. Some established combination analgesics, which have shown significant results, act by combining analgesics which have a different mechanism or site of action or combining drugs which have short-acting and long-acting effects [28].

Morphine is one of the drugs of choice for treatment of pain but the development of tolerance is usually considered to be an obstacle in its therapeutic uses. Tolerance can be defined as a state of adaptation in which exposure to a drug induces receptor changes that result in a decreased biological efficacy of drug action [29]. In simple pharmacological terms it is a shift to the right in the dose-response curve. Besides, a higher dose of drug is required over time in maintaining the same level of analgesia. 

Tolerance is a feature of all opioid medications [28]. Drugs such as morphine are tolerated by the body over time. The longer that morphine is taken, the more tolerant the body becomes to the drug, so larger and larger doses are needed to relieve pain and the medication becomes less effective with time. Tolerance is a normal phenomenon commonly associated with the administration or prolonged use of drugs, and repeated administration of morphine may lead to decreased analgesic effectiveness in humans and animals. Tolerance can develop after administration of several types of opioids delivered via various routes, doses and administration schedules [28]. 

In the previous study [15], we have studied the antinociceptive effects of MG in the hot-plate test. The study showed that MG did exert its analgesic effect through the activation of opioid receptors. Until now, there are no studies which have been design to investigate the effects of combining MG and morphine. Perhaps combinations of MG and morphine that offer analgesic synergism would allow the reduction of the required dosage which gives optimum analgesic effects with low incidence of tolerance. In this study, we investigated the analgesic effects of morphine in combination with the natural phytocompound mitragynine. The tolerance development was assessed and compared to the group that was treated with morphine alone (Figure 2).

Desensitization and molecular changes of opioid receptors result in the development of tolerance. Opioid receptors belong to the G-protein-coupled receptor family (GPCRs) [28]. When morphine, MG or an opioid analgesic is bound to the receptor, the associated G protein becomes ‘activated’. The activation of the receptor decreases the activity of adenylyl cyclase, resulting in a decrease in the production of cyclic adenosine monophosphate (cAMP). This leads to an increase in the efflux of K^+^ and cellular hyperpolarization and a decrease in the influx of Ca^2+^ and lower intracellular concentrations of free Ca^2+^. The overall consequences are a decrease in the neuronal release of neurotransmitters and this result in analgesia [30]. 

The pharmacological action of morphine involves binding to and activation of opioid receptors in the peripheral, as well as the central nervous system [31]. The binding of morphine with opioid receptors results in analgesic effects, known as antinociception. This can be shown as a delayed pain perception in experimental studies [32,33]. Morphine acting on opioid receptors via the Gi/Go classes of G-proteins inhibits adenylyl cyclase which results in reduced intracellular cAMP formation [20,34]. This results in inhibition of neuronal excitability and synaptic transmission mediated via cAMP [35]. 

In our previous study [15], the ED_50_ of mitragynine was 31.8 mg/kg. On that basis, 15 and 25 mg/kg were chosen as the dose used in this study indicating that lower dosee should be used for a longer duration of treatment. Besides, during our acute combination study, 15 and 25 mg/kg exerted a synergistic analgesic effect when combined with morphine in a single dosage (data not shown). In the present study, the administration of morphine increased the latency time in the hot-plate test on day 1. The result was statistically significant when compared to the control group. It was proven that morphine at 5 mg/kg exerts analgesic effects and it may be because of delayed pain perception via the activation of opioid receptors (Figure 2). However, the latency time was reduced on day 5 to day 9 which was the end of the experiment. It is noted that the reduction in latency time was significant when compared to the latency time on day 1. This suggested that there was a reduction in analgesic activity of morphine which may show as a faster pain perception. This also demonstrated that morphine has developed tolerance whereby at this stage, a higher dosage of morphine is required to give the same level analgesic effects to the pain stimulus as day 1. Even though during the experimental period day 5 to day 9, the mitragynine (15 and 25 mg/kg b.wt) groups showed a reduction of latency time, the reduction was not significant when compared to the respective group latency time of day 1.

Mitragynine (15 and 25 mg/kg b.wt) was found to increase the latency time in mice. Our results coincide with a report by Matsumoto *et al.* [12,13]. Mitragynine also showed antinociceptive effects on thermal stimuli. From our previous findings, the antinociceptive action of mitragynine may involve opioid receptors as the effect of mitragynine was antagonized by intraperitoneal injection of naloxone, norbinaltorpimine and naltrindole. This suggests that the compound has antinociceptive effects and it acts via the activation of opioid receptors. The related previous reports also proved that opioid receptors were involved in the analgesic action of mitragynine [12,13,36].

Meanwhile, in the group treated with the combination of mitragynine and morphine, latency time of the combinational group (especially 5 Mor + 25 mg/kg MG) was significantly improved compared to control group showing the effect of treatment with morphine alone. Interestingly, the analgesic effect of this combination was seen throughout the experimental study from day 1 until day 9. Moreover, the combination of mitragynine and morphine may prevent the development of tolerance as compared to a single repeated morphine administration. Furthermore, this result indicates that the concurrent treatment has sustained the analgesic action of morphine, besides the action of mitragynine itself.

The present study has shown that prolonged exposure to morphine reduced the analgesic effects of this drug which can be expected to lead to the development of drug tolerance. Previous findings have suggested that the combinations of opioid analgesics and other analgesics can be used advantageously to control pain. The use of several combinations of potent opioids was suggested to reduce the side effects of opioid treatment, to improve analgesia and to reduce opioid tolerance [16,17]. This study proved that the combination of morphine and mitragynine is a good combination which is suggested to improve analgesia. One theory of opioid tolerance involves changes in opioid receptors. It has been well known that opioids such as morphine have been shown to differ in their ability to desensitize opioid receptors [37,38]. The theory stated that receptors undergo changes that results in decreased receptor activation with prolonged exposure to opioids [37]. 

Present findings indicate a possible relationship of receptor desensitization and drug tolerance. Morphine have been proved to desensitize opioid receptor which result in the decreased of analgesic. Together in combination with mitragynine, the desensitization of opioid receptor may be prevented. This results in the enhancement of the latency time and maintaining of the analgesic effects throughout experimental study.

In another theory of tolerance, with continuing opioid exposure, activity of cAMP signaling gradually recovers and increases (upregulation) above normal levels which is in contrast with the normal antinociceptive effects of opioids. Thus, an increase in cAMP levels was suggested to represent a cellular model of tolerance and dependence [39]. cAMP regulates the expression of many genes via a conserved gene promoter element CRE (cAMP response element) [21]. The cAMP response element binding (CREB) is a transcriptional factor that binds to CRE in the promoter region of target genes and modulates their expression [22,23]. CREB is a constitutively expressed transcription factor activated through the cAMP pathway. The activation of CREB after chronic treatment might suggest that CREB may form the cellular basis of tolerance and dependence [26,27,40,41]. The activation of cAMP will lead to the activation of CREB, and *vice versa*. 

In the present study, we investigated the expression of cAMP by immunoassay kit to detect the development of tolerance at the molecular level. The thalamus and cortex part have been separated and analyzed for the expression of cAMP. The reason of choosing the cortex and thalamus is because these parts are involved in the pain pathways and perception of pain [42,43]. In a study by Simone *et al*. [44], tolerance did enhance the expression of cAMP in the thalamus and cortex using f MRI. The molecular evaluation of tolerance also has been investigated further in the assessment of CREB by the western blotting method using whole brain tissue lysates. In our finding, the chronic treatment of morphine significantly increased the levels of cAMP and CREB protein. This finding is in line with previous research findings [24,45] which proved that chronic morphine exposure effectively increased cAMP levels and also influence the increase of CREB protein expression. Thus, it is noteworthy that an opioid-induced increase in cAMP and CREB level is inter-linked to the development of tolerance to morphine treatment [24]. Therefore, it was suggested that the alterations in CREB activity following chronic morphine treatment were caused by the activation of cAMP pathway which reflected through the upregulation of cAMP. However, the combination regimen as well as mitragynine (15 and 25 mg/kg b.wt) alone did not increase the level of expression of cAMP and CREB, suggesting that the combination of mitragynine with morphine decreased (down regulated) the activation of the cAMP pathway which subsequently prevents the development of tolerance. This agrees with the second theory of tolerance. The results of cAMP and CREB protein expression level were in line with the hot-plate antinociceptive findings (Figure 3 and Figure 4). 

Toxicity analysis of combinational treatment was performed to investigate the non-toxic nature of the treatment in the experimental model. Liver and kidney function analysis is practiced as a usual screening parameter to detect adverse effects on the liver and kidney of treatment. The liver is the most central organ in the metabolism of various drugs and other synthetic substances. Liver cell destruction shows its effects mostly as important in the liver cell membrane permeability, which results in the leaking out of tissue content into the blood stream [46]. In vital organs, cell membrane destruction/damage is followed by release of a number of cytoplasmic enzymes to the blood, a phenomenon that provides the basis for clinical diagnosis. Altered serum activities of aspartate trans-aminase (AST), alanine transaminase (ALT) and γ-glutamyltransferase (GGT) are of clinical and toxicological importance, being indicative of vital tissue damage by toxicants or specific disease conditions [47]. Except for the 25 MG group that showed a significant change in ALT levels, the present findings showed that combination treatment of mitragynine and morphine treatment results in no significant change in the activities of serum enzymes which can be stated that the single drug as well as combination treatment is non-toxic to the mammalian system (Figure 5).

Nephrotoxicity is one of the major side effects of drug therapy in clinical practice, frequently leading to acute renal failure. Kidneys maintain the optimum chemical composition of body fluids by acidification of urine and removal of metabolite wastes such as urea, uric acid and creatinine. In renal toxicity, the concentrations of these metabolites increase in blood. Kidney injury can be assessed by the presence of urea and creatinine in the blood stream. Therefore, measuring the levels of both creatinine and urea in blood gives an impression of renal function. Various studies indicate that, since urea levels may also increase as a result of increased protein catabolism, creatinine is generally used as a more reliable marker for renal function [48]. In the present study, there was a significant change in creatinine levels for the higher concentration combination treatment (5 Mor + 25 MG b.wt), but no significant change in the level of urea. Based on the histopathological observation, it has concluded that the treatment did not have any adverse effects on kidney when the H&E staining of kidney shown that the architecture of kidney is preserved (data not shown). These findings demonstrated that non-toxic nature of combination treatment (Figure 6) however, further analysis is required to investigate the fate of the compounds excretion via the kidneys. 

## 3. Experimental

### 3.1. Drugs and Chemicals

Morphine was purchased from Sigma-Aldrich Co., (St. Louis, MO, USA). The diagnostic kits for liver and kidney function tests were procured from Roche Diagnostics (Berlin, Germany). All other chemicals/reagents used in the study were of analytical grade. 

### 3.2. Plant Materials

Mature leaves of *Mitragyna speciosa* Korth. (MS) were collected from natural sources of different places around Peninsular Malaysia. Authentication of plant material was carried out by Mr Kamarulizwan Kamaruddin, a qualified botanist from the Faculty of Forestry, Universiti Putra Malaysia where vouchers (ATS: 001) have been deposited in the herbarium for future reference. 

### 3.3. Isolation of Mitragynine from Mitragyna speciosa

Mitragynine was isolated from the leaves of *Mitragyna speciosa* according to the methods described by Houghton and Ikram, [6] with a minor modification. One kg leaves of MS material was cleaned and dried with constant temperature at 45 °C overnight before being ground into powder form. It was then macerated with methanol for 72 h. The mixture was then filtered to remove the insoluble particles and obtain the methanolic crude extract. The extracts further were evaporated using a rotary evaporator (Eyela, Tokyo, Japan) at a high temperature (55 °C). 

The extracts was added with 5% (v/v) sulphuric acid and stirred overnight. The mixture was filtered and a clear yellow solution known as the acidic filtrate was obtained. The acidic filtrate was then added with sodium carbonate and stirred until it turns into a dark grey (pH 11) basic filtrate. To separate the alkaloids from the crude extract, chloroform was added to the mixture and the fraction was decanted in a separating funnel. Three layers were produced: aqueous, salt and chloroform layer. The chloroform layer was then simply separated and filtered for further processing. The chloroform fraction was mixed with anhydrous sodium sulphate and evaporated to yield 0.73% (w/w) of crude extract. The major alkaloid isolated by silica gel chromatography eluting with diethyl ether was identified as mitragynine by standard spectroscopic methods (^1^H-NMR, ^13^C-NMR) [15]. Overall, the yield of active constituent mitragynine was 0.087% (w/w) of fresh weight of the leaves.

### 3.4. Animals

Healthy male ICR mice weighing between 25–35 g were housed and kept at 25–30 °C in the Animal Center, Faculty of Medicine and Health Sciences (FMHS), Universiti Putra Malaysia. Animals were divided into six groups (n = 7) in a temperature-controlled room. They were maintained under standard laboratory conditions with natural dark and light cycle. They were allowed free access to food (standard commercial food pellets) and tap water *ad libitum*. Animals were acclimatized for at least one week to adapt to the laboratory prior to test. All animal experimental procedures were approved (Approval no: UPM/FPSK/PADS/BR-UUH/00307) by Institutional Animal Care and Use Committee (IACUC) FMHS, Universiti Putra Malaysia (UPM).

### 3.5. Hot-Plate Test

The antinociceptive activity of test compounds are determined by exposing animals to potentially painful stimuli such as heat or electric shock and measuring either the times it takes them to respond to the stimuli or the intensity with which they respond. If the time it takes the animal to respond to the stimulus is prolonged following test compound or drug administration, then it is presumed that the compound and drugs altered perception of the painful stimulus. In this study, the hot-plate test was used for assessment of latency time. Briefly, mice were placed on a stainless steel surface which was maintained at the temperature of 50 ± 0.2 °C (UgoBasile Model No: 7280, Comerio VA, Italy) and latency time was recorded. The latency time is defined as the time when the mice were placed in the heat surface until the occurrence of nociceptive responses such as licking, shaking or jumping was observed. The cut-off time of 50s was used to prevent tissue damage. 30 min prior to treatment the nociceptive threshold was measured and the latency time has been used as the pre-drug latency for each test animal. The latency time was calculated as:

Latency time (s) = time when the mice showed nociceptive responses(s) − pre drug latency (s)

### 3.6. Experimental Design

Mice were randomly assigned equally into six groups of seven mice. The animals were tested for pre-drug latency before used for experiment where mice with pre-drug latency below five seconds or more than 20 s were eliminated from the present study. The purpose of this step was to avoid bias for mice with extremely short or long pre-latency responses that could contribute to some variances in the analysis. Groups assigned were as follows: Control (NS); mitragynine 15 mg/kg; mitragynine 25 mg/kg; morphine 5 mg/kg; combination of morphine 5 mg/kg + mitragynine 15 mg/kg and combination of morphine 5 mg/kg + mitragynine 25 mg/kg b.wt (Table 1). Administration of all test drugs was performed intraperitoneally (i.p) and all treatments were given 15 min before the hot-plate test. The treatment was given repetitively, once a day for 9 days (total experimental period). The antinociceptive response using hot-plate test was measured before and continuously after daily injections for 2 h at 15 min time intervals. All mice were sacrificed on day 9 after the hot-plate test. Blood from each mouse was collected and kept at 4 °C overnight before being centrifuged at 3,000 rpm for 15 min to separate the serum for liver function tests (LFT) such as alanine aminotransferase (ALT), aspartate aminotransferase (AST) and γ-glutamyltransferase (GGT) and kidney functions tests (KFT) including urea and creatinine. Brain tissues were dissected out and snap freeze under liquid nitrogen and kept at −80 °C for biochemical analysis.

**Table 1:** Illustration of each group and its respective treatment.

Group	Initial	Description
Control	NS	Normal saline
Treatments	15MG	15 mg/kg mitragynine
	25MG	25 mg/kg mitragynine
	5Mor	5 mg/kg morphine
	5Mor + 15MG	5 mg/kg morphine + 15 mg/kg mitragynine
	5Mor + 25MG	5 mg/kg morphine + 25 mg/kg mitragynine

### 3.7. Measurement of cAMP Level

The frozen dissected brain tissues were thawed and homogenized with hand held homogenizer (Polytron PT1600E, Berlin, Switzerland). The homogenate was centrifuged at 15,000 × *g* for 10 min. The supernatant was extracted with water-saturated diethyl ether to remove the acid. Finally, the level of cAMP was quantified in the aqueous extract using an enzyme immunoassay kit (Cellbio, San Diego VA, USA) according to the manufacturer’s instructions.

### 3.8. Immunoblotting Analysis

The frozen dissected brain tissues were thawed and homogenized with a hand held homogenizer (Polytron PT1600E). The homogenate was centrifuged at 13,000 rpm for 10 min at 4 °C and the supernatant was collected and stored at −80 °C for further analysis. Later on, the pellet was resuspended and filtered through 0.4 µm gauze and centrifuged filtrate for three times in 5,000 rpm for 5 min. The filtrate was added with 2× solubalization buffer before used. The nuclear fraction was used for the measurement of CREB protein expression. The protein concentration was measured using Bovine Serum Albumin (BSA) protein assay. Test samples and standards were run on 10% SDS-PAGE, and then immuno-blotted with primary antibody (anti-CREB ab 1:500) and with goat anti rabbit horseradish peroxidase (HRP)-conjugated secondary antibody (1:3,000 Serotec). The membrane was developed using enhanced chemiluminescent substrate (Thermo Scientific, Kalamazoo, MI, USA) for visualization of CREB protein. The developed membrane was exposed using GelDoc (Chemismart-3126 WL/26MX, Courbevoie, France). Protein bands were analyzed using Image J (version 1.0) software. The blot was reprobed with β-actin antibody to ensure equal protein loading.

### 3.9. Statistical Analysis

The results were presented as means ± SEM after analyzed by using Statistical Package for the Social Sciences (SPSS) version 16.0. One Way ANOVA for group comparison followed by *post hoc* Multiple Comparison test were applicable for inter-group comparisons. The significance of difference was defined as *p* values <0.05.

## 4. Conclusions

In conclusion, we have demonstrated that combinations of mitragynine and morphine increase the analgesic effects of morphine in prolonged exposure, and reduce/prevent the development of morphine tolerance and this is speculated to be mediated by the down regulation of the cAMP pathway. In addition, the combination treatments induced slight (based on the toxicological parameters) side effects during the experimental period. We suggest further research of these compounds to elucidate their biological properties through determination of their specific molecular mechanism of action.
